# Effects of pulmonary fissure completeness on major outcomes in children after video-assisted thoracoscopic congenital lung malformation lobectomy

**DOI:** 10.1186/s12887-022-03527-4

**Published:** 2022-08-01

**Authors:** Jin-Xi Huang, Qiang Chen, Song-Ming Hong, Jun-Jie Hong, Hua Cao

**Affiliations:** 1 Department of Cardiac Surgery, Fujian Branch of Shanghai Children’s Medical Center, 966 Hengyu Road, Fuzhou City, Fujian Province, China; 2 Fujian Children’s Hospital, 966 Hengyu Road, Fuzhou City, Fujian Province, China; 3 College of Clinical Medicine for Obstetrics and Gynecology and Pediatrics, Fujian Medical University, 966 Hengyu Road, Fuzhou City, Fujian Province, China; 4 Fujian Key Laboratory of Women and Children’s Critical Diseases Research, Fujian Maternity and Child Health Hospital, 966 Hengyu Road, Fuzhou City, Fujian Province, China

**Keywords:** Pulmonary fissure completeness, Video-assisted thoracoscopic surgery, Children, Lobectomy, Complications

## Abstract

We performed a single-centre retrospective analysis using data from databases that were prospectively maintained in our centre between January 2019 and September 2021. Patients were divided into two groups based on the degree of pulmonary fissure completeness (PFC), using the fissure development scoring system. Patients with grades 2 or 3 PFC were considered to have incomplete pulmonary fissures and were included in Group A, and patients with grades 0 and 1 were considered to have complete pulmonary fissures and were included in Group B. The differences in demographics, perioperative characteristics and clinic outcomes between the two groups were evaluated. Multivariate logistic regression analysis was performed. A total of 213 patients with congenital lung malformation (CLM) underwent video-assisted thoracoscopic lobectomy. There were 30 patients in Group A and 183 patients in Group B. Our data showed that compared with Group B, Group A had a higher incidence of complications, especially Clavien-Dindo grade II and grade III complications. The degree of PFC was significantly correlated with the length of chest tube drainage and postoperative hospital stay. Multivariate logistic regression analysis showed that the degree of PFC could be used to predict the incidence of postoperative complications.

**Conclusions**

The degree of PFC is a predictor of the incidence of complications after thoracoscopic lobectomy in children with CLM.

## Background

Congenital lung malformation (CLM) is the general term for a group of malformations of the lung, and congenital pulmonary airway malformation (CPAM) and pulmonary sequestration (PS) are the most common types of CLM [1, 2]. The pathological characteristic of CLM is abnormal pulmonary morphogenesis during the pseudoglandular phase of foetal lung development [3]. The incidence of CLM is 1:2500 [4, 5]. Lesions associated with respiratory distress require resection, but asymptomatic lesions can be monitored. The removal of asymptomatic lesions can prevent infections and malignant transformation [6, 7]. Video-assisted thoracoscopic surgery (VATS) has become a commonly used minimally invasive technique for the treatment of various thoracic diseases in children [8–10]. The thoracoscopic treatment of CLM has also been widely reported and has been shown to have many advantages over thoracotomy [11, 12]. However, despite advances in surgical techniques and perioperative care, the incidence of postoperative complications remains 11–18% [3, 13, 14]. The degree of pulmonary fissure completeness (PFC) is an important issue that paediatric thoracic surgeons often encounter during VATS lobectomy. The current evidence suggests that incomplete lobar fissures can increase the surgical difficulty and the likelihood of a prolonged air leakage and a prolonged hospital stay [15, 16]. The effect of PFC on other major hospitalization outcomes has also been discussed [17]. Most of the articles cited above report studies on adult thoracoscopic surgery. Navallas et al. reported the impact of pulmonary fissure dysplasia on children after lung surgery, including open surgery and thoracoscopic surgery [16]. However, few articles have mentioned the effect of PFC on the incidence of postoperative complications after thoracoscopic lobectomy in children. In this study, we quantified the degree of PFC and assessed the impact on primary outcome during hospitalization and to assess its predictive effect on the incidence of complications after thoracoscopic lobectomy in children with CLM.

## Materials and methods

We retrospectively analysed the clinical data of CLM patients who underwent VATS lobectomy in our hospital from January 2019 to September 2021 obtained from a prospectively maintained database of medical records at our centre. Inclusion and exclusion criteria: Target diseases were surgically treated CLM, including CPAM and intralobar PS. Only patients treated with standardized lobectomy with complete VATS were included. Extralobar PS was excluded because it is relatively simple to perform, is not affected by the completeness of the lung fissure and does limited damage to normal lung tissue. To eliminate the influence of different surgical procedures, other surgical procedures, such as wedge resection and segmentectomy, were also excluded to avoid confounding bias. Data on perioperative characteristics and outcomes were extracted for further analysis.

The objectives of our study were the major outcomes and postoperative complications associated with VATS lobectomy. Each patient was followed for more than 6 months after surgery. We recorded the outcome data in this study as follows.

Baseline patient characteristics, including age, sex, and weight, were recorded. Preoperative comorbidities included respiratory tract infection, chest wall abnormality and malnutrition. We defined preoperative respiratory infection (PRI) as the presence of one or more infectious diseases, such as aspiration pneumonia, lung abscess, and respiratory bacterial/fungal infection. Malnutrition was defined as a weight-for-age below minus 3 standard deviations of the mean. Chest wall abnormality include pectus carinatum and pectus excavatum. The intraoperative variables analysed included lesion location, operative time, and intraoperative blood loss. Two surgeons with 8 years of clinical experience independently quantified the degree of PFC intraoperatively, using the fissure development scoring system reported by Lee et al. [18]. The evaluation of the degree of fissure development was based on the improved evaluation model, which was based on the original PFC classification [19].

Grade 0: Complete fissure with fully separated lobes;

Grade 1: Complete visceral cleft with more than 70% completeness of the interlobar fissure within the lung parenchyma;

Grade 2: Partly evident visceral cleft with 30%–70% completeness of the interlobar fissure within the lung parenchyma;

Grade 3: No evident fissure line with less than 30% completeness of the interlobar fissure within the lung parenchyma.

Based on the cut-off proposed by Lee et al. and the method proposed by Li et al. [18, 20], patients were divided into two groups according to the degree of PFC. Patients with grades 2 or 3 PFC were considered to have incomplete pulmonary fissures and were included in Group A, and patients with grades 0 and 1 were considered to have complete pulmonary fissures and were included in Group B. The Clavien-Dindo classification system divides postoperative complications into five incidence levels [21]; however, it has not been used to evaluate complications of a large number of paediatric thoracoscopic surgeries. We conducted a statistical analysis of postoperative complications by referring to the classification method in the study by Khan and Pio et al. [13, 22]. The postoperative hospital stay was calculated from the day of operation to the day of discharge. The chest tube was removed when there was no air leakage and the chest volume was less than 1 mL/kg/d [23]. VATS lobectomy was performed using a three-portal approach under bronchial occlusion. All patients received intravenous patient-controlled analgesia to control their postoperative pain, a standard protocol of 2.0 μg/kg of sufentanil and 100 mL of physiologic saline was used with the speed of 2.0 mL/h for the first postoperative 48 h. This protocol is equivalent to 1 μg/kg/d of sufentanil.

All statistical analyses were completed with SPSS 22.0 software (IBM Corporation, Armonk, NY, USA). Continuous data are presented as the means with SDs, while categorical data are presented as patient frequency and percentages. In the univariate analysis, we used Pearson’s χ^2^ to compare categorical variables and Student’s t-test to compare the mean values of continuous variables, for the data with *n* < 5, we use Fisher's Exact Test to compare categorical variables. Multivariate logistic regression analysis was used to determine the independent risk factors for major inpatient conditions. The binary data with *P* < 0.05 in the univariate analyses were incorporated into the logistic regression model, and the accuracy of the model was adjusted with the Hosmer–Lemeshow test and the C statistic. Finally, through multivariate logistic regression analysis, ORs with 95% CIs were obtained. Statistical significance was set at *P* < 0.05.

## Result

In total, 213 patients underwent VATS lobectomy during the study period. There were 30 patients in Group A and 183 patients in Group B. The baseline patient characteristics and outcomes are summarized in Table 1. This cohort consisted of 125 male (58.7%) and 88 female (41.3%) patients with a mean age of 5.8 ± 4.0 months and a body weight of 8.0 ± 1.7 kg. There was no significant difference in preoperative general characteristics between the two groups. The mean operative time was 73.2 ± 18.2 min, and Group A had a higher percentage of right upper lobe (33%, *p* < 0.001), a longer operative time (96.9 ± 14.9 min, *p* < 0.001), and more intraoperative blood loss (24.3 ± 10.9 ml, *p* < 0.001) than Group B. No procedures were converted to thoracotomy in either group. Most patients were diagnosed with CPAM (159 patients, 74.6%), followed by intralobular PS (54 patients, 25.4%). There were no pathological differences between the two groups. The length of chest tube drainage was 3.0 ± 1.5 days, and the postoperative hospital stay duration was 4.6 ± 1.5 days. Group A had a longer length of chest tube drainage and a longer hospital stay than Group B. There were no reoperations and no deaths.

**Table 1:** Patient characteristics and outcomes

	A group	B group	Total	*P*-value
Age(months)	4.2 ± 0.9	6.1 ± 4.3	5.8 ± 4.0	0.054
Gender(male)	19(63.3%)	106(57.3%)	125(58.7%)	0.153
Weight(kg)	6.6 ± 1.6	8.2 ± 1.5	8.0 ± 1.7	0.098
Preoperative respiratory infection	3(10%)	17(9.2%)	20(9.4%)	0.203
Malnutrition	1(3.3%)	3(1.6%)	4(1.9%)	0.112
Chest wall abnormality	1(3.3%)	6(3.2%)	7(3.3%)	0.243
Location				
Right upper lobe	10(33.3%)	16(8.7%)	26(12.2%)	*p* < 0.001
Right middle lobe	1(3.3%)	21(11.5%)	22(10.3%)	
Right lower lobe	6(20%)	57(31.1%)	63(29.6%)	
Left upper lobe	5(16.7%)	26(14.2%)	31(14.6%)	
Left lower lobe	8(26.7%)	63(34.4%)	71(33.3%)	
Operation time (minutes)	96.9 ± 14.9	69.3 ± 15.5	73.2 ± 18.2	*p* < 0.001
Blood lost (ml)	24.3 ± 10.9	15.2 ± 3.5	15.4 ± 11.8	*p* < 0.001
Pathological parameters				
Congenial pulmonary airway malformation	21(70.0%)	138(75.4%)	159(74.6%)	0.333
Intralobar pulmonary sequestration	9(30.0%)	45(24.6%)	54(25.4%)	
Overall complication	8(26.7%)	28(15.3%)	36(16.9%)	0.011
Clavien-Dindo grade I	3(10%)	19(10.4%)	22(10.3%)	0.198
Subcutaneous emphysema	1	11	12	
Atelectasis requiring sputum aspiration	2	3	5	
Wound infection	0	3	3	
Transient phrenic palsy	0	2	2	
Clavien-Dindo grade II	3(10%)	6(3.3%)	9(4.2%)	*p* < 0.001
Prolonged air leak > 2 days	2	6	8	
Pneumonia	1	0	1	
Clavien-Dindo grade III	2(6.7%)	3(1.6%)	5(2.3%)	*p* < 0.001
Atelectasis requiring a bronchoscope	1	2	3	
Chylothorax	1	1	2	
Length of chest tube drainage (days)	3.5 ± 3.4	2.0 ± 0.8	3.0 ± 1.5	0.032
Length of hospital stay(days)	5.2 ± 3.5	3.9 ± 0.7	4.7 ± 1.5	0.020

Note: Data presented as n (%) unless otherwise stated

Postoperative complications occurred in 36 of the 213 patients, with a total incidence of 16.9% (Table 1). The incidence of Clavien-Dindo grade I complications was 10.3% (*n* = 22), that of grade II complications was 4.2% (*n* = 9), and that of grade III complications was 2.3% (*n* = 5). The 3 most common complications were subcutaneous emphysema (*n* = 12, 5.6%), prolonged air leak > 2 days (*n* = 8, 3.8%), and atelectasis requiring sputum aspiration (*n* = 5, 2.3%). There were 8 (26.7%) postoperative complications in Group A, which was significantly higher than the value in Group B (*n* = 28, 15.3%), and statistically significant differences are reflected in the incidence of grade II and grade III complications. Incidence of grade I complications: Univariate analysis revealed 5 significant influencing factors, as shown in Table 2. The C-statistic for our logistic regression model confirmed that PRI (OR 2.33, 95% CI 1.90–5.32 *P* = 0.01) was an independent predictor of grade I complications. Incidence of grade II complications: Table 2 shows the five risk factors identified in the univariate analysis. A C-statistic indicated that an incomplete pulmonary fissure (OR 2.08, 95% CI 0.69–4.56, *P* < 0.01) and an operation duration > 120 min (OR 2.18, 95% CI 1.84–5.41, *P* < 0.01) are independent risk factors for morbidity. Incidence of grade III complications: There were five risk factors identified in the univariate analysis, and we identified PRI (OR 2.87, 95% CI 2.05–6.22, *P* < 0.01), incomplete pulmonary fissure (OR 2.61, 95% CI 2.09–5.76, *P* < 0.01), and operation duration > 120 min (OR 2.91, 95% CI 2.55–8.15, *P* < 0.01) as independent risk factors.

**Table 2:** Multivariate analysis of risk factors for postoperative complications

Factors	OR	95% CI	*P*-value
Clavien-Dindo grade I			
Gender (male vs female)	1.89	1.11–2.81	0.30
Preoperative respiratory infection	2.33	1.90–5.32	0.01
Malnutrition	1.32	0.90–1.78	0.41
Chest wall abnormalities	1.11	0.69–1.55	0.33
Pulmonary fissure completeness	1.85	1.10–3.50	0.08
Duration of surgery (> 120 vs ≤ 120 min)	1.20	1.07–2.07	0.16
Clavien-Dindo grade II			
Preoperative respiratory infection	1.22	0.97–1.55	0.39
Malnutrition	1.39	0.88–2.01	0.51
Chest wall abnormalities	1.52	1.21–1.87	0.42
Pulmonary fissure completeness	2.08	1.69–4.56	< 0.01
Duration of surgery (> 120 vs ≤ 120 min)	2.18	1.84–5.41	< 0.01
Clavien-Dindo grade III			
Preoperative respiratory infection	2.87	2.05–6.22	< 0.01
Malnutrition	1.26	0.86–1.68	0.34
Chest wall abnormalities	1.22	1.05–1.93	0.39
Pulmonary fissure completeness	2.61	2.09–5.76	< 0.01
Duration of surgery (> 120 vs ≤ 120 min)	2.91	2.55–8.15	< 0.01
All complications			
Gender (male vs female)	1.77	1.32–2.17	0.55
Preoperative respiratory infection	2.39	1.83–4.59	< 0.01
Malnutrition	1.11	0.78–1.57	0.81
Chest wall abnormalities	1.39	0.90–1.98	0.19
Pulmonary fissure completeness	2.55	1.87–5.44	< 0.01
Duration of surgery (> 120 vs ≤ 120 min)	2.38	1.66–5.12	< 0.01

**Fig. 1:**
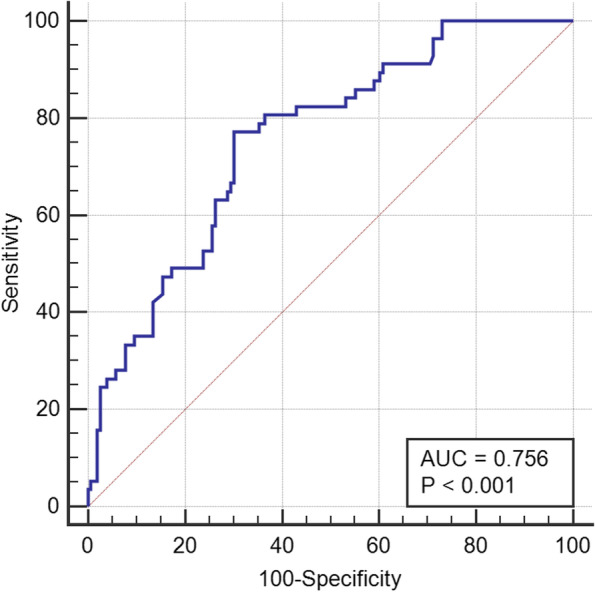
Receiver-operating-characteristic analysis on discriminative power of multivariate logistic-regression model for predicting overall morbidity of postoperative complications. C-statistic presented as area under curve

A multivariate logistic regression model containing all 6 significant clinical parameters was established. The C-statistic for our logistic-regression model was 0.76, as shown in Fig. 1. Finally, we determined that PRI (OR 2.39, 95% CI 1.83–4.59, *P* < 0.01), incomplete pulmonary fissure (OR 2.55, 95% CI 1.87–5.44, *P* < 0.01), and operation duration > 120 min (OR 2.38, 95% CI 1.66–5.12, *P* < 0.01) are independent risk factors for overall complication-related morbidity after adjusting the multivariate logistic regression analysis for confounding effects.

## Discussion

The degree of PFC is a common problem encountered by thoracic surgeons during pulmonary surgery, and it is one of the factors influencing the outcomes during and after the operation. There are several possibilities for assessing fissures: computerized tomography (CT) scans; endobronchial systems assessing collateral flow and perioperative methods. A meta-analysis [24] of 31 related articles showed: The position of the fissures exhibits high variability, sometimes a fissure is missing completely (usually the horizontal one), the prevalence of incomplete fissures ranged from 19.2% to 77% (mean 48.7), 17.4% to 87% (mean 55.4), and 48.3% to 89% (mean 69.8) for left oblique, right oblique, and horizontal fissures, respectively. In our data, the location of the lesion in group A was significantly more frequent in the right upper lobe (33.3% vs 8.7%), this is similar to the results of other literatures’. Another possible reason for this result is: although some lesions located in the left side were found with complete fissures, but it is also possible that there is a incomplete horizontal fissures in another side, which can not be confirmed from the surgery.

In our data, the operation duration in Group A was longer than that in Group B, and this was considered to be caused by the relatively greater number of surgical procedures. The dissection of the lung parenchyma inevitably took more time in Group A patients. In addition, some surgical time was also spent determining the pulmonary lobe boundary and wound haemostasis. In terms of intraoperative bleeding, the amount of blood lost in Group A was greater than that in Group B, mainly because there was still a small amount of bleeding when the lung parenchyma was separated. It is worth noting that there were 3 patients in Group A who lost more than100 ml of blood due to vascular injury. Although there was no conversion to thoracotomy after haemostasis during VATS, such bleeding can often be fatal. When a fissure is incomplete, the surgeon cannot expose the vessels from the fissure, and taking a single-direction VATS lobectomy approach may result in damage to the posterior tissues. For example, especially in lobectomies of the lower lobe, the pulmonary artery that needs to be removed is located directly behind the lower bronchi. When the connective tissue between the lower bronchi and the pulmonary artery is removed, the damage to the pulmonary artery may have serious consequences. In our study, Group A had a longer length of chest tube drainage than Group B. Pleural drainage is associated with injury to the lymph nodes, microlymphatic vessels and hilar lung parenchyma [25]. During the process of separating the lung parenchyma with LigaSure, thermal damage is inevitable, which may lead to increased exudate from the wound surface and perihilar wounds. Another reason may be that the operation duration and single-lung ventilation duration in Group A were inevitably longer than those in Group B, which may lead to more pulmonary oedema and lung exudation [26, 27]. We discharged patients on the second day after chest tube removal, which was also the main reason the postoperative length of hospital stay in Group A was longer than that in Group B.

Cases of Pneumothorax after thoracic surgery were mainly due to parenchymal air leakage. Three asymptomatic pneumothorax cases were observed in Group A, while 1 was observed in Group B. In patients in Group A, the pulmonary parenchyma overlying the artery needed to be split. Due to the small space in the thorax, it is difficult to separate the pulmonary parenchyma using Endo-GIA in children with CLM, therefore, the separation is usually performed by cautery or with LigaSure, which may cause air leakage. However, all of these patients were cured by increasing the length of chest tube drainage, and no further invasive or secondary surgery was performed.

Postoperative complications were the focus of this study, and our data showed that the overall incidence of complications was significantly correlated with the degree of PFC. The Clavien-Dindo classification system, which has acceptability and reproducibility, can be used to classify all postoperative complications into five grades from mild to severe. This classification system has also been applied by many researchers to the statistical analysis of complications after thoracoscopic surgery [13, 28, 29]. It is reassuring that there were no serious grade IV or V complications in this cohort. The incidence of grade II and III complications, such as pneumothorax, aggravated pneumonia, atelectasis requiring bronchoscopy and chylothorax, was significantly correlated with the degree of PFC, indicating that the incidence of postoperative complications increased as the pulmonary fissure dysplasia worsened. Multivariate logistic regression analysis showed that the degree of PFC was an independent factor influencing the main outcomes during hospitalization, including length of stay, operative duration, intraoperative blood loss, and length of chest tube drainage. We believe that these data and analyses can help surgeons, because the degree of PFC can be preliminarily assessed with preoperative computerized tomography [17]. This preliminary assessment can be used to inform the the surgeon’s preoperative decision regarding which surgical to use and the surgeon’s communication with the patient’s family, informing them of the possible postoperative complications, which constitute an important part of preoperative conversations with family members [30].

In addition, for young surgeons, this preliminary assessment may assist in the selection of their patients when they are early in the learning curve or the development of a teaching program for the VATS technique. It is also possible to preplan the treatment of serious complications. We suggest that the degree of PFC should be considered when informing the parents of the children about the incidence of potential complications and selecting patients who are suitable for treatment by a surgeon who is early in the learning curve. This constitutes a multifaceted effort to keep this particular group of patients as safe as possible.

The shortcomings of this study are the inherent limitations of a single-centre retrospective population-based study, although the multivariable logistic regression analyses involved as many clinicopathological variables as possible in an attempt to minimize the risk of bias from potential confounding factors, selection bias may still have complicated our results. Second, the sample in our study was relatively small, which may limit the analytical power. Such as, even there is no statistically significant difference in ages between group A and B, *p* value is 0.054. The association between age and the degree of pulmonary fissure completeness has not been reported and there is a lack of relevant studies, which may require larger sample sizes and prospective trials to draw conclusions.

## Conclusion

Our study shows that the degree of PFC is a categorical predictor of the incidence of postoperative complications. Incomplete pulmonary fissure is significantly associated with a prolonged length of chest tube drainage and hospital stay, and also with the overall morbidity of complications, especially Clavien-Dindo grade II and III after thoracoscopic lobectomy in children with CLM.

## Data Availability

The data that support the findings of this study are available on request from the corresponding author. Requests to access these datasets should be directed to hjx7072@126.com.
